# A bedside staffing model with perfusionists for pediatric extracorporeal membrane oxygenation (ECMO) at a high-volume center★

**DOI:** 10.1051/ject/2025040

**Published:** 2026-03-13

**Authors:** Christopher Nemeh, Caitlin Cain-Trivette, Nicholas Schmoke, Caleb Varner, Michael Brewer, Eunice Clark, Holly Ream, Diana Vargas Chaves, Tarif Choudhury, Steven Stylianos, William Middlesworth, Eva W. Cheung

**Affiliations:** 1 Division of Pediatric Surgery, Department of Surgery, Columbia University Vagelos College of Physicians and Surgeons / NewYork-Presbyterian Morgan Stanley Children’s Hospital 3959 Broadway New York NY 10032 USA; 2 Department of Perfusion, NewYork-Presbyterian Morgan Stanley Children’s Hospital 3959 Broadway New York NY 10032 USA; 3 Department of Nursing, NewYork-Presbyterian Morgan Stanley Children’s Hospital, Columbia University Medical Center 3959 Broadway New York NY 10032 USA; 4 Division of Pediatric Neonatology, Department of Pediatrics, Columbia University Vagelos College of Physicians and Surgeons / NewYork-Presbyterian Morgan Stanley Children’s Hospital 3959 Broadway New York NY 10032 USA; 5 Division of Pediatric Cardiology, Department of Pediatrics, Columbia University Vagelos College of Physicians and Surgeons / NewYork-Presbyterian Morgan Stanley Children’s Hospital 3959 Broadway New York NY 10032 USA; 6 Division of Pediatric Critical Care and Hospitalist Medicine, Department of Pediatrics, Columbia University Vagelos College of Physicians and Surgeons / NewYork-Presbyterian Morgan Stanley Children’s Hospital 3959 Broadway New York NY 10032 USA

**Keywords:** Extracorporeal membrane oxygenation, Perfusion, Pediatrics, Critical care, Workforce

## Abstract

*Background*: Extracorporeal membrane oxygenation (ECMO) in pediatric patients requires monitoring by specialists to optimize outcomes. Practice variability exists among pediatric ECMO centers across the country. We present a bedside pediatric ECMO staffing model with perfusionists that combines personnel expertise and technology. *Methods*: At our institution, ECMO care is provided in three intensive care units across one floor. Our primary bedside ECMO provider consists of pediatric perfusionists who provide 24/7 coverage of ECMO patients via remote monitoring and hourly bedside rounding. Neonatal and pediatric ECMO patients are supported using the Cardiohelp System^TM^, which uses Spectrum Medical Quantum Elite Workstation and Variable Input Patient Electronic Records (VIPER) software that remotely delivers ECMO circuit parameters and alarms digitally to a monitor in a workroom and mobile devices. ECMO education and skills are reinforced via dedicated didactic and simulation sessions by an ECMO program coordinator. We compared institutional complication rates to other centers tracked by ELSO. *Results*: From 2017 to 2023, 289 cannulations were performed, consisting of a total of 62,742 cumulative ECMO hours, of which 92% of that time there were simultaneous ECMO patients. This rounding model has institutional mortality and complication rates that are comparable to ELSO rates. *Conclusion*: We describe a bedside ECMO staffing model with perfusionists, in contrast to ECMO specialists seen at other institutions. The complication and mortality rates are comparable to ELSO rates, suggesting the safety of this model. Further exploration regarding resource utilization and costs is warranted.

**Abbreviations**
ECMO: Extracorporeal membrane oxygenation
ICU: Intensive care unit
AV: Arteriovenous
VIPER: Variable Input Patient Electronic Records
ECPR: Extracorporeal cardiopulmonary resuscitation
ELSO: Extracorporeal Life Support Organization

## Introduction

Pediatric ECMO is challenging and requires extensive resources and trained professionals for optimal safety and efficacy [1, 2]. In many pediatric ECMO centers, ECMO specialists with extensive training provide the day-to-day monitoring of ECMO patients and are typically staffed at the bedside of each ECMO patient [3]. Most ECMO specialists are respiratory therapists or nurses who have completed additional ECMO training and are stationed at the bedside to monitor ECMO patients in a 1:1 ratio [3, 4]. The practice variability of ECMO monitoring and staffing across pediatric centers is mainly due to resource utilization and availability of personnel. Although perfusionists are advanced practitioners with ECMO experience, most centers use trained ECMO specialists due to multiple factors, including potentially reducing costs, perfusionist shortage, and greater availability and flexibility of nurses and respiratory therapists [3, 4]. We use remote monitoring for ECMO patients, which is a novel concept that has been described with adequate response time to troubleshoot circuitry issues in adults [5, 6]. The goal of our staffing model is to develop an effective and safe system for monitoring ECMO patients that uses hospital resources, technology, education, and personnel to its advantage [3, 4]. We present a bedside model of ECMO staffing that leverages the combination of expertise by perfusionists and advanced technology to remotely monitor multiple pediatric ECMO patients simultaneously.

## Materials and methods

**Figure 1:**
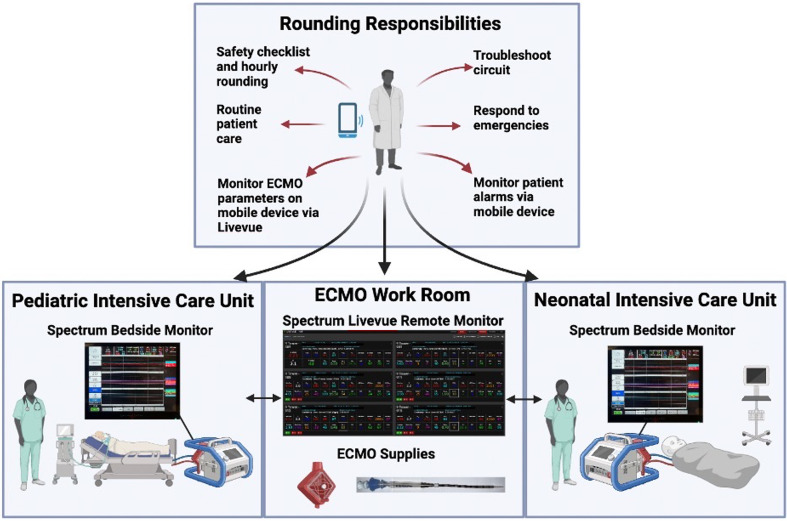
Graphical description of our novel ECMO staffing model. (Created in BioRender. Cain, C. (2024) BioRender.com/s22z994).

Our institution is a pediatric quaternary care high-volume ECMO center in an urban environment. There are, on average, 48 pediatric ECMO cannulations per year since this model was implemented. Our staffing model uses one perfusionist per 12-hour shift to monitor all ECMO patients on a single floor (Figure 1). In our model, there are 12 perfusionists who cover ECMO. ECMO care is provided in three intensive care units (ICUs) – a pediatric cardiac ICU (14 beds), a neonatal cardiac ICU (17 beds), and a pediatric med/surg ICU (13 beds), which are all located on the same floor. ECMO cannulation may occur in various areas across our hospital due to the availability of mobile ECMO carts. Cannulations are performed by pediatric cardiothoracic surgeons or pediatric general surgeons. If a patient is cannulated outside of the three ECMO ICUs, they are moved to one of the three ECMO units and monitored by a bedside perfusionist for the first 24 h post-cannulation, followed by remote monitoring and an hourly rounding model by the perfusionist team.

All neonatal and pediatric ECMO patients are supported using the Cardiohelp System^TM^ and either an HLS 5.0 disposable and 1/4″ AV loop with a shunt in order to meet the minimum flow rate through the oxygenator or an HLS 7.0 disposable and 3/8″ AV loop. The Cardiohelp HLS system is manufactured by Getinge USA Sales, LLC, located at 1 Geoffrey Way, Wayne, NJ 07470, USA. Of note, the 1/4″ AV loop circuits allow for a manifold to assist with access, especially for neonates. Each ECMO circuit is connected to a Spectrum Medical Quantum Elite Workstation, which uses Variable Input Patient Electronic Records (VIPER) software and Livevue (Spectrum Medical’s web-based near real-time remote access tool) that remotely delivers ECMO circuit parameters [6]. The Quantum Elite Workstation with VIPER software and Livevue is manufactured by Spectrum Medical, whose USA headquarters are located at 481 Munn Road, Suite 180, Fort Mill, SC 29715, USA. The workstation integrates parameters directly from the Cardiohelp System^TM^, such as revolutions per minute, circuit pressures, and temperature, while directly measuring parameters such as flows, saturations, PaO2, and PCO2. The workstation also integrates with the Phillips Intellivue monitor to access patient parameters such as temperatures, blood pressures, pulse oximeter readings, and near-infrared tissue saturations. These variables are sent to a hospital-based server where they can be accessed for remote viewing facilitated by VIPER and LiveVue. Patients’ vital signs, ECMO circuit parameters, and alarms are remotely monitored using Philips^©^ technology and Livevue, which delivers critical notifications to a computer screen in the perfusionists’ workroom and their mobile devices via push notifications. LiveVue pulls its information from the Spectrum Medical monitoring system, whose data comes from a variety of probes on the ECMO circuit.

The perfusionist rounds hourly on each circuit, performs a checklist of tasks, and is readily available to troubleshoot and assist in patient-related tasks (i.e., daily rounding, patient turning, physical therapy, etc.). The checklists broadly include, but are not limited to verification of patient, medical record number, evaluating the pump, zeroing the pump, alarm parameters set, hand crank being available, electrical connections being intact, pump tubing with no kinks and banded where appropriate, checking for clots and air in tubing, oxygenator evaluation with packing intact and no defects, gas being on, bubble detector being operational, and shunt closed (if applicable), cleaning all touch and control surfaces, ECMO settings verified (sweep, flow, etc.), VIPER alarms set appropriately, temperature alarms, and handoff updated.

Pediatric ECMO fellows are available 24/7 and are the first contact for ECMO consults, standbys, and extracorporeal cardiopulmonary resuscitation (ECPR). ECPR simulations are performed bimonthly, consisting of all the multidisciplinary teams involved during a live cannulation. ECPR simulations are immediately followed by a debriefing session that enhances collaboration among all the participants during cannulation.

A designated ECMO program coordinator provides training to the frontline ICU nurses through ECMO simulations, didactic education days, and workshops. The ECMO program coordinator at our institution is a nurse practitioner. A one-time ECMO didactics course is required for all ICU nurses who work on the ECMO floors. Didactics consist of lectures, scenarios, simulations conducted by chief perfusionists, and a multiple-choice quiz based on the Extracorporeal Life Support Organization (ELSO) red book, 6th edition. A score of 80% or higher is required to pass the course.

To determine the safety and efficacy of our model, we compared the mortality and complication rates of our institution with ELSO rates. The total number of ECMO hours was calculated to quantify the total time cannulated during the study period. Simultaneous ECMO run hours were also calculated to stratify how frequently one perfusionist needed to monitor more than one ECMO patient.

## Results

**Figure 2:**
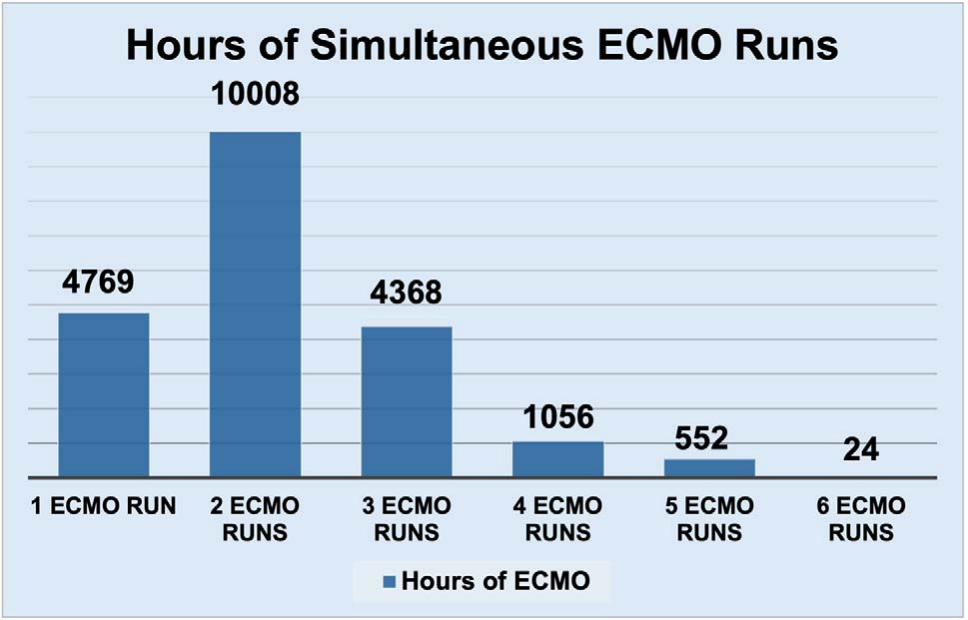
Hours of simultaneous ECMO runs.

From 2017 to 2023, 289 cannulations were performed, comprising 62,742 cumulative ECMO hours. Simultaneous ECMO runs were evaluated, and the hours were calculated (Figure 2), of which 92% of the total time was spent with simultaneous ECMO runs. Our mortality and complication rates are compared to the ELSO national rates as shown in Tables 1 and 2. Mortality rate from 2013 to 2017, prior to the institution of the model, was 44.4%, which is comparable to after the staffing model was implemented.

**Table 1:** NewYork-Presbyterian Morgan Stanley Children’s Hospital vs. ELSO mortality data.

Patient Cohort (9/2017–12/2023)	Adjusted mortality rate	ELSO adjusted mortality rate
Neonatal Patients	46.70%	39.50%
Pediatric Patients	47.40%	39.80%

**Table 2:** NewYork-Presbyterian Morgan Stanley Children’s Hospital vs. ELSO neonatal and pediatric complication data.

Complication detail	Unadjusted rate neonatal	ELSO unadjusted rate neonatal	Unadjusted rate pediatric	ELSO unadjusted rate pediatric
Any mechanical complication	33.80%	31.50%	28.10%	26.40%
Oxygenator failure	3.08%	5.26%	9.18%	5.13%
Raceway rupture	0.00%	0.05%	0.00%	0.06%
Other tubing rupture	0.00%	0.09%	0.00%	0.22%
Pump malfunction	1.54%	0.70%	0.51%	0.67%
Heat exchanger malfunction	0.00%	0.22%	0.00%	0.18%
Clots: hemofilter	0.00%	2.68%	0.00%	2.05%
Clots: circuit component clots	6.15%	12.00%	7.65%	8.00%
Air in circuit	6.15%	2.74%	2.04%	2.97%
Cracks in pigtail connectors	0.00%	0.08%	0.00%	0.13%
Cannula problems	9.59%	10.20%	3.9%	8.59%
Circuit change	6.15%	12.60%	8.16%	9.31%
Clots and air emboli	1.54%	1.02%	0.51%	0.45%
Thrombosis/Clots: circuit component	4.62%	11.10%	7.14%	8.09%
Any hemorrhagic complication	26.20%	19.50%	25.50%	21.20%
Cannulation site bleeding	10.80%	12.60%	11.70%	12.80%
Surgical site bleeding	12.30%	7.32%	13.80%	8.08%
Hemolysis (PFH > 50 mg/dl)	21.50%	15.30%	9.69%	11.00%

PFH = plasma free hemoglobin.

## Discussion

Our model was implemented since 2017. Other centers typically use bedside ECMO specialists or perfusionists for each cannulated patient. We have one pediatric perfusionist who can monitor multiple cannulated patients simultaneously. We always have three perfusionists on-call to cover ECMO activities and operating room needs. One perfusionist is responsible for monitoring ECMO patients. A second perfusionist assists in any additional ECMO initiations, including ECPR, transports, and circuit exchanges, so we have a level of redundancy for patients already on support. The rounding ECMO perfusionist rounds every hour so they can review patients’ labs/notes and take meal breaks in between rounds. Our ECMO staffing model utilizes perfusionists who conduct hourly rounds; however, institutions considering a similar approach should carefully evaluate their workforce capacity and staffing resources to ensure feasibility and sustainability. Protocols and safety measures implemented at our institution make this model effective, including modern technology, ECMO education and simulations, and an ECMO program coordinator. Our coordinator is a nurse practitioner who serves as a liaison between the ECMO team and the ICU teams. We also conduct biweekly ECMO team meetings and debriefing sessions after cannulations. Although our model uses perfusionists, a similar model could potentially be carried out with ECMO specialists.

Our mortality and complication rates are comparable to the ELSO rates. Our mortality rates are not adjusted for; however, a literature review of pediatric ECMO has mortality rates ranging from around 30 to 50% which is comparable to our mortality rate [7, 8]. The mortality rates from 2013 to 2017 are 44.4%, which is comparable to the mortality rates after implementation of the rounding model. The complications described in our institution and ELSO are not uniquely related to ECMO staffing models. The duration of ECMO with multiple patients cannulated significantly exceeds the duration of solo runs. This suggests our model is safe and effective for ECMO staffing and monitoring even with simultaneous ECMO runs. Monitoring by nurses is effective at other institutions; however, it is frequent that these ECMO specialists will require consultation with perfusionists when initiating cannulations and troubleshooting ECMO circuitry [4]. A perfusionist model provides experienced caregivers for ECMO monitoring; however, this must be leveraged with cost and availability [9]. The cost burden of a perfusion-led system may be more substantial than that of an ECMO specialist-led model with equivocal outcomes [4, 10]. Other centers have used ICU-run models in adults due to provider availability and cost reduction, with equivocal outcomes [11]. Institutions across the country utilize various ECMO staffing models, and the choice of one system over the other should be individualized at each institution [9]. While resource utilization and cost are important, optimizing patient care and outcomes is the top priority regardless of the staffing model [9].

### ECMO education and simulations

Due to the complexity of ECMO, it requires frequent training and simulations for providers to remain proficient and quickly troubleshoot issues. Our ECMO program coordinator organizes simulation and didactic education days for ICU nurses. All nurses working on ECMO units must take the course and pass a multiple-choice exam to ensure that they understand and escalate high-risk scenarios when they occur. These didactics allow the nurses to be formally trained with an ECMO curriculum. In addition, we have multidisciplinary ECMO simulations to enhance teamwork and evaluate all cannulation processes for improvement. Several studies stress the importance of continuing education and ECMO simulations to improve patient safety and outcomes [2, 12, 13]. Simulations assemble all involved participants during cannulations, whether controlled or during ECPR. During simulations, a mock code is performed where the medical team begins CPR and activates the ECMO team. This allows the team to practice transitioning from CPR to ECPR. A CPR manikin is used with a plastic neck that can be cut down on in order to simulate a live cannulation. Debriefing after these sessions is crucial to assessing for improvements and system flaws that may not be readily apparent [14]. Even within our institution, debriefs following the ECPR simulations enhance collaboration and allocation of specific tasks when a real ECPR occurs. Debriefings occur immediately after the session, and the goal is to familiarize the team with CPR, ECMO cannulation, and the associated problems that may arise before, during, and immediately after cannulation. ECMO education is invaluable to ensuring that staff are prepared to deal with common and rare complications that can occur during an ECMO run, which is essential in our model’s efficiency and safety [2, 12, 15, 16].

### Technology

Our technology allows ECMO circuit parameters and alarms to be sent to a workroom and mobile devices. This allows perfusionists to monitor multiple ECMO patients simultaneously. The perfusionist can set desired ranges such that if a circuit parameter falls out of range, the system will notify the perfusionist via the monitor connected to LiveVue and their mobile devices [6]. The LiveVue system is fully customizable, so perfusionists can input different parameters from the circuit and lab parameters. Having all ECMO patients on the same floor allows for a quick response time by the perfusionist to troubleshoot alarms and emergencies as they arise. We exclusively use the Cardiohelp System^TM^ for neonatal and pediatric ECMO, which simplifies circuit components and allows for the ease of delivery of circuit parameters. VIPER records ECMO circuit parameters, enabling the perfusionist to track and identify changes when troubleshooting issues with the ECMO circuit. The VIPER system utilizes formulas and algorithms, referred to as clinical guidance, that can be customized by the ECMO team to identify specific issues and alert personnel. Examples in our practice, which combine inputs from several different systems, are recirculation on venovenous ECMO and poor distal perfusion during femoral venoarterial ECMO with distal perfusion catheters. Perfusionists are automatically alerted to the presence potential issues based on changes in the measured parameters. Automating these notifications helps to improve the consistency of care between perfusionists aiding in the ability to monitor several simultaneous ECMO patients.

## Conclusions

Pediatric ECMO staffing requires multidisciplinary care to ensure patient safety and optimal outcomes. We describe an ECMO staffing model with perfusionists, in contrast to ECMO specialists who are typically used in high-volume pediatric ECMO centers. Our model involves remotely monitoring pediatric ECMO patients with one pediatric perfusionist per 12-hour shift, even when multiple patients are simultaneously on ECMO. All ECMO units are on the same floor, which allows our perfusionists to cover multiple patients simultaneously and quickly respond to issues when they arise. Our duration of ECMO with multiple patients cannulated significantly exceeds the duration of solo runs. This, combined with the low incidence of adverse events, demonstrated by having comparable numbers to ELSO rates, suggests this model’s potential safety even with multiple patients on ECMO. Our staffing model has several factors that enable it to work efficiently, including an ECMO program coordinator, integrated technology for alerting perfusionists, and frequent ECMO education and simulation for all participating providers.

## Data Availability

All data obtained are included in the manuscript, and further information is available on request.
